# Adapting Grapevine Productivity and Fitness to Water Deficit by Means of Naturalized Rootstocks

**DOI:** 10.3389/fpls.2022.870438

**Published:** 2022-05-24

**Authors:** Emilio Villalobos-Soublett, Nicolás Verdugo-Vásquez, Irina Díaz, Andrés Zurita-Silva

**Affiliations:** ^1^Centro de Investigación Intihuasi, Instituto de Investigaciones Agropecuarias INIA, La Serena, Chile; ^2^Centro de Investigación Raihuén, Instituto de Investigaciones Agropecuarias INIA, San Javier, Chile

**Keywords:** *Vitis vinifera*, naturalized rootstocks, *V. berlandieri* × *V. rupestris*, hydric behavior, deficit irrigation, gas exchange, water use efficiency

## Abstract

Climate change effects are unbalanced in all regions and cultivars linked to the wine industry. However, the impact of extreme weather events, such as drought and rising global temperatures, highlight the potential vulnerability in plant productivity, phenology, and crop water requirements that affect quality and harvests. Among adaptative measures for grapevine cultivars in existing or new winegrowing areas, the use of tolerant rootstocks to abiotic stress has been regarded as a mid-term strategy to face emerging constrains. The aim of this study was to compare naturalized or autochthonous rootstocks influence over grapevine cultivar performance and to characterize their response to deficit irrigation conditions. Data was collected from Cabernet Sauvignon and Syrah grafted plants for over 3 growing seasons (2018–2021) from a hyper-arid experimental field in Vicuña, Chile. Morpho-physiological parameters were determined throughout seasons and combinations where significant effects from rootstocks, irrigation treatment, and cultivar were observed over A_n_ and g_s_, thus modifying CO_2_ assimilation and intrinsic Water Use Efficiency (WUE_i_). Primary productivity and yield were also modified by rootstock depending upon cultivar hydric behavior. Interestingly, cluster and berry traits were unaffected despite how water productivity and integral water stress were modulated by rootstock. In both cultivars, it was observed that trait responses varied according to the irrigation conditions, rootstocks, and their respective interactions, thus highlighting a relative influence of the rootstocks in the processes of adaptation to the water deficit. Moreover, harvest date and acidity were modified by deficit irrigation treatment, and rootstocks did not modify phenological stages. Adaptation of grapevines to expected lower water availability might be improved by using suitable tolerant rootstocks, and maturity index can be modified through irrigation management.

## Introduction

Among environmental constraints, water scarcity is probably the most important threat all over the world (Jury and Vaux, 2005; Kijne, 2006; Berger et al., 2010), compromising agricultural production at several latitudes (FAO, 2018). Rapidly, the possibilities to generate and to manage new water sources for agriculture will be limited, and the instability of water resources will not only be detrimental to crop productivity, but will also generate substantial socioeconomic impacts (Postel, 2000; Polade et al., 2017). Considering the competition for water use among agriculture, human consumption, and industrial sectors, crop water demands could double by 2050, whereas the availability of freshwater is predicted to drop by 50%, owing to global climate change (CC; Gupta et al., 2020). Viticulture production is not an exception, since future projections of CC-driven changes or “climate crisis” suggest a lack of water to maintain current levels of production in all regions of the world, which will particularly impact Mediterranean ecosystems (Hannah et al., 2013). Furthermore, suitable zones for grapevine production based on temperature may be greatly affected in the Mediterranean regions (Santillán et al., 2019). Viticulture adaptation to CC in these regions (as most crops do) will require integrated strategies and major adaptive levers to cope with water availability and grapevine productivity and an increase in evapotranspiration that encompass different levels of organization: the crop (cultivar and rootstock), the cropping system (management techniques used), and the farming system, including farmers (del Pozo et al., 2019; Naulleau et al., 2021).

A recent systematic study identified current knowledge to evaluate adaptation strategies in the main vineyards worldwide (Naulleau et al., 2021), whose findings were as follows: (1) evaluation of a combination of adaptation strategies provides better solutions for adapting to CC; (2) multi-scale studies allow local constraints and opportunities to be considered; and (3) only a small number of studies have developed multi-scale and multi-lever approaches to quantify feasibility and effectiveness of adaptation (Naulleau et al., 2021). For instance, ecophysiological studies have contributed to maximize the use and productivity of water, i.e., irrigation technification, regulated deficit irrigation (Hsiao et al., 2007), soil mulch, and optimization of the orchard density and architecture (Ripoche et al., 2010). However, in mid-term, it will be necessary to incorporate increasing productivity, fruit quality and disease tolerance, criteria of water productivity [WP; g (MS)/mm (H_2_O) transpired], and water stress tolerance in cultivars and rootstock breeding (Warschefsky et al., 2016; Simonneau et al., 2017; Gupta et al., 2020). Thus, a water shortage scenario will demand to assess interaction effects of cultivar x rootstock x environment and their impacts in fruit attributes associated with its quality (Ibacache et al., 2016; Cochetel et al., 2017; van Leeuwen et al., 2019; Villalobos-González et al., 2019).

Under water deficit conditions, perennial plants display a continuum of mechanisms for dealing with low water availability between two edges: 1) drought evasion, which is found in species bearing high stomatal sensitivity (near-isohydric), or 2) drought tolerance, found in species bearing low stomatal sensitivity response to the ambient (near-anisohydric) and functional and morphological traits toward adaptation such as osmo-regulation (Schultz, 2003; Blum, 2011; Gupta et al., 2020). Stomatal closure is linked to systemic signaling from the roots rather than the shoot, as evidence that root physiological status plays a key role in controlling the shoot behavior (Lawlor, 2002). Traditionally, the plant hormone abscisic acid (ABA) plays an essential function as a phytochemical signal involved in the shoot-root communication, because of drop in soil water potential (Zhang et al., 2006). It is also considered the most prominent player in drought stress, directly affecting stomatal conductance (gs) at the guard cell level (Gupta et al., 2020). The physiological and molecular mechanisms driving the ABA effects are yet to be summarized (Gambetta et al., 2020).

In grapevines, the hydraulic and biochemical modes of stomatal regulation are interdependent, making a strict division between them extremely challenging both theoretically and experimentally (Medrano et al., 2003; Ollat et al., 2016; Buckley, 2019; Gambetta et al., 2020). Moreover, the relative contribution of these mechanisms is still unknown and likely dependent on genotype and environment (Lovisolo et al., 2016; Coupel-Ledru et al., 2017; Hochberg et al., 2018; Dayer et al., 2020). It has also been demonstrated that leaf hydraulic conductance (K_leaf_) was downregulated by exogenous ABA, with strong variations depending on the genotype (Coupel-Ledru et al., 2017). Interestingly, variation between isohydric and anisohydric genotypes correlated with K_leaf_ sensitivity to ABA, with K_leaf_ being unresponsive to exogenous ABA in the most anisohydric genotypes (Coupel-Ledru et al., 2017). Recent work suggests that all genotypes regulate stomatal conductance to protect against more severe damage, such as petiole or leaf cavitation and leaf shedding (Dayer et al., 2020). However, it is not clear to what extent differences in regulation of vine water use between cultivars result from innate genotypic differences or environmental factors (Hochberg et al., 2018). Likewise, grapevines appear to almost always operate within a “safe” margin of water potentials in which stem cavitation is extremely rare (Charrier et al., 2018). Still, the exact vine mortality thresholds are still unknown. Thus, numerous gaps remain in understanding of what really configures a drought-adapted grapevine cultivar, making it difficult to robustly address future climate challenges (Gambetta et al., 2020). Also, unraveling mechanisms to explain how the regulation of aerial (cultivar) drought tolerance could be enhanced by a root-driven feedback regulation, where the role of rootstock might be crucial to determine such responses and sustain productivity, pointing toward an integrative approach that digs into the complexity of cultivar x rootstock x environment interaction (Franck et al., 2020).

Most of the worldwide vineyards are grafted on commercial rootstocks which are hybrids of mostly three species, namely, *Vitis berlandieri, V. riparia*, and *V. rupestris*, that were developed before 1930 from American *Vitis* species to control phylloxera damage (Serra et al., 2014; Berdeja et al., 2015). Rootstocks also provide support for cultivation under challenging soil conditions, including the presence of nematodes and insects, high salinity or active lime, and drought (Meggio et al., 2014; Serra et al., 2014; Walker et al., 2014). Limited long-term information on rootstock effects over yield and its components are available. Nevertheless, it is established that the response is primarily associated with the vigor level conferred to the scion by the rootstock (Dry and Loveys, 1998). This influences bud fruitfulness and vine productivity (Satisha et al., 2010; Ibacache et al., 2016). To sustain grapevine productivity and quality in CC warming, an increase for irrigation water will occur, generating big freshwater demands, considering the low adoption of measurements of RDI or PRD. Therefore, agricultural adaptation efforts that anticipate these multiple possible effects in Mediterranean agroecosystems are needed (Hannah et al., 2013; Wolkovich et al., 2018). By assessing the current effects of CC on *V. vinifera*, it is key to understand the plasticity associated with the ability to uptake water from soil in the continuous root/vine/environment (Santillán et al., 2019; van Leeuwen et al., 2019).

Although scion-rootstock interactions in drought tolerance have been studied (Serra et al., 2014; Tomás et al., 2014; Bianchi et al., 2020), the diversity of rootstocks adapted to dryer conditions is limited. Recent studies in rootstock effects were evaluated, and differences in fruit yield, pruning weight, budburst, fruit set, bunch weight, berry weight, berry diameter, and rachis weight between nine rootstocks in semiarid conditions were determined along with their effects on nutrient uptake (Ibacache et al., 2016, 2020). Therefore, grapevine rootstocks will undoubtedly play a fundamental role in the adaptation to future CC, especially to water shortage (Serra et al., 2014; Ollat et al., 2016; Delrot et al., 2020) and to improve WP, but mechanisms driving these processes are still elusive. In a grapevine meta-analysis contrasting stomatal conductance in response to water availability, rootstock genotype explained the greatest contribution to variability (19.1%) followed by the scion genotype (16.2%) (Lavoie-Lamoureux et al., 2017). Moreover, the effect of soil water-holding properties was analyzed and showed a scion-dependent effect which was dominant over rootstock effect in predicting gs values. Overall results suggest that a continuum exists in the range of stomatal sensitivities to water stress in *V. vinifera*, rather than an isohydric—anisohydric dichotomy, which is further enriched by diversity of scion-rootstock combinations and interactions with soils and intensity of water deficits (Levin et al., 2020).

In Chile, naturalized rootstocks were collected from arid regions of Northern Chile (Milla-Tapia et al., 2013) and studied in response to water deficit. Some selected genotypes induced significantly higher tolerance for morpho-physiological traits irrespective of scion and seasons associated with higher root growth at early stages (Franck et al., 2020). Further transcriptomic analysis was performed, determining that major differences in transcriptional behavior occurred at root level, suggesting scion-driven transcriptional regulation in response to water deficit (Franck et al., 2020). Despite the importance of grapevine phenology, studies on the effect of rootstock on the development of phenological stages are scarce in literature and have been carried out in few varieties and under different edaphoclimatic conditions (Loureiro et al., 2016; van Leeuwen and Destrac-Irvine, 2017; van Leeuwen et al., 2019).

To understand the effects and expected impacts of CC warming due to water deficit on grapevine productivity and fruit maturity, we conducted a multi-rootstock approach using two contrasting cultivars in regard to hydric behavior grafted on selected naturalized rootstocks to assess the array of response as study model for understanding adaptive responses that might confer better drought adaptation to specific clone/rootstock combinations on vegetative and fruit expression in hyper arid environment.

## Materials and Methods

### Plant Material, Drought Stress Conditions, and Physiological Measurements

The field experiment was conducted during three growing seasons (2018/19, 2019/20, and 2020/21) at an experimental vineyard located at the Vicuña Experimental Center belonging to the Instituto de Investigaciones Agropecuarias (INIA) (30°02′S, 70°41′W, 630 m above sea level; Coquimbo Region, Chile). The climate of the area is classified as hyper-arid, with an average daily temperature of 16.1°C and a mean annual rainfall of 100 mm that concentrates in winter (June–September). The vineyard soil is a sandy loam alluvial Entisol and has a flat topography (<1%). The soil holds moderate depth (>50 cm) with no physical restrictions for root growth. A pit was made, determining that the roots were concentrated in the 30 cm depth. From 0 to 30 cm depth, a soil sample was taken, obtaining the following composition: sand (54.1%), slime (28%), clay (17.85), field capacity (11.2% v v^−1^), permanent wilting point (5.2% v v^−1^), pH value (7.3, calcareous soil), organic matter (1.5%), and electrical conductivity (2.3 dS m^−1^ in saturated paste). Cultivars Cabernet Sauvignon (CS, near-isohydric) and Syrah (Sy, near-anisohydric) were grafted onto two naturalized genotypes (R32 and R70) selected in northern Chile for their tolerance to water deficit (Bavestrello-Riquelme et al., 2012; Milla-Tapia et al., 2013; Franck et al., 2020), to commercial tolerant rootstock Ruggeri140 (140Ru), and to self-grafted vines (SG). Both varieties grafted onto rootstocks were assigned in a completely randomized design at planting.

The grapevines were planted during spring 2015 with a spacing of 1 ×2.5 m within north-south oriented rows, trained on a vertical shoot positioning (VSP) trellis system, formed in unilateral cordon, and cane pruned to a Guyot system leaving about 6–8 buds per vine. Due to the low rainfall that was characteristic during the seasons (<100 mm), it was necessary to apply water through irrigation. Thus, grapevines were drip irrigated using one irrigation line per row with emitters supplying water at a rate of 4 l h^−1^ spaced at 1 m (1 emitter per plant) located on the surface, 15 cm from the trunk. Weather variables (air temperature, relative humidity, solar radiation, precipitation, wind speed, and wind direction) were measured at 15 min time during the season, using an automatic meteorological station (Adcon Telemetry, A730, Klosterneuburg, Austria) located near the experimental vineyard (30 m). This information was used to calculate the reference evapotranspiration (ET0) using the Penman–Monteith model (Allen et al., 1998). Then, the actual evapotranspiration (ETa) was calculated by adjusting the ET0 by the crop coefficient (Kc) corresponding to each phenological stage, using the value described by Jara-Rojas et al. (2015). The reference evapotranspiration during the three seasons varied between 792.4 and 797.7 mm (September–April).

The experimental design consisted of two water regime treatments per cultivar with three replicates (blocks) of five grapevines each to cope for soil variability along the vineyard: full irrigation (T0) and 50% deficit irrigation (T1) *via* a drip irrigation system in both cultivars that was randomly distributed within rows. The 50% deficit irrigation was considered since the observed decline of precipitation over central Chile has been greatly accentuated by an uninterrupted sequence of dry years since 2010, with annual rainfall deficits ranging between 25 and 45% (Garreaud et al., 2020). Field trial received a standard agronomic management used in commercial vineyards in terms of irrigation, fertilization, pruning, pest, and disease management in each growing season.

Nutritional content of the soil was described elsewhere (Verdugo-Vásquez et al., 2021a). In brief, nutritional content of the soil at the beginning of the study was 40 mg kg^−1^ of available N, 8 mg kg^−1^ of available P, 105 mg kg^−1^ of available K, 8.2 meq 100 g^−1^ of available Ca, 2.0 meq 100 g^−1^ of available Mg, 22.0 mg kg^−1^ of available Fe, 7.0 mg kg^−1^ of available Mn, 6.3 mg kg^−1^ of available Zn, 11.4 mg kg^−1^ of available Cu, and 1.8 mg kg^−1^ of available B. The fertilization program consisted of applications of N, P_2_O_5_, and K_2_O (90, 50, 70 kg ha^−1^, respectively) during each growing season, *via* irrigation (fertigation), dividing the mentioned doses in each irrigation (~3 per week) during spring and early summer. It was only fertilized with N, P, and K because the other nutrients were at adequate levels. The sources of commercial fertilizers used were “Ultrasol Nit One 25,” “potassium sulfate,” and “Ultrasol Pro P.” Through foliar analyses carried out in veraison, it was determined that there were no deficiencies or excess of nutrients during the development of the study. Therefore, fertilization was not a limitation.

Physiological trait measurements included the stem water potential (Ψ_stem_) taken from fully mature and healthy leaves (two per replicate) located in the center of the west facing vine canopy between 12:00 and 15:00 h (Solar noon; Coordinated Universal Time UTC−3) from November to March using a pressure chamber (PMS Instrument Co., model 600, Corvallis, Oregon, USA). For these measurements, the leaves were covered with completely hermetic aluminum foil bags for at least 1 h before the measurement. Then, leaves were cut and immediately placed in the chamber. Moreover, to describe the accumulated effect of the deficit irrigation treatments between rootstocks, the water stress integral (SIΨ) was calculated as follows (Myers, 1988):


$$SI\Psi =\;\left|\sum \left(\Psi_{stem}-c\right)n\right|$$

Where Ψ_stem_ is the average stem water potential for any interval (MPa day), c is the maximum value of Ψ_stem_ during the season, and n is the number of days in each interval (Moriana et al., 2007).

Also, the stomatal conductance (g_s_; mol H_2_O m^−2^s^−1^) and net assimilation rate (A_n_; μmol CO_2_ m^−2^ s^−1^) were measured on fully sunny, developed, and healthy leaves located in the mid center of the canopy facing west using a portable infrared gas analyzer (LI−6400, LICOR Inc., Lincoln, Nebraska, USA) equipped with a 6 cm^2^ transparent leaf chamber. Environmental conditions in the leaf chamber were photosynthetically active radiation ≥ 2,000 μmol photon m^−2^ s^−1^, a molar air-flow rate setting at 500 μmol s^−1^, and a concentration of 400 μmol s^−1^ CO_2_ that was kept constant by a CO_2_ injector system provided by the manufacturer. These measurements were taken on the same days and times when the Ψ_stem_ was measured. Also, the intrinsic water use efficiency was calculated from the ratio between A_n_ and g_s_ (A_n_/g_s_; WUE_i_, μmol CO_2_ mol H_2_O^−1^).

### Grapevine Phenology Determinations

The phenology observations were made using the scale proposed by Coombe (1995) and followed the procedure described by Verdugo-Vásquez et al. (2016). Briefly, 3 main phenological stages were observed (budburst, flowering, and veraison) through observations made every 5–7 days, expressing the dates of occurrence of the phenological stages in day of the year (DOY) for each cultivar and season. Additionally, the duration of the growth cycle from budburst to veraison was determined by calculating the number of days elapsed between both phenological stages (expressed in days).

### Berry Maturity Measurements

From post-veraison (4–15 days after veraison) to harvest (defined when the berries reached between 22 and 23°Brix of total soluble solids), berry samplings (4 dates) were carried out following the procedure described by Verdugo-Vásquez et al. (2018). In each of the sampling dates, berry maturity parameters (total soluble solids, total acidity, and pH) were determined according to the Organization of Vine and Wine (OIV) protocol (International Organisation of Vine and Wine (OIV), 2021). With the evolution curves of total soluble solids, the day of the year when the berries reached 22.5°Brix was determined, recording this day as the harvest date.

### Productivity Traits Measurements

At harvest, all bunches of the replicates were manually harvested and weighed in a digital weight scale, recording yield by vine (kg vine^−1^) and the number of bunches per vine. The bunch weight (g) was determined by dividing the yield by the number of bunches per plant. A sample of three clusters per replicate was taken to the laboratory where the following variables were determined: N° berries per bunch, berry weight, rachis length, rachis weight, and caliber. Vines of each replicate were manually pruned in winter, and the pruning weight (kg vine^−1^) was determined. Based on yield and pruning weight obtained, the Ravaz index was calculated as the ratio between yield and pruning weight, representing the balance between vine reproductivity and vegetative activity for each season. Scion trunk circumference (cm) was measured at the end of each season (May) at 30 cm above the ground using a metric tine. Water productivity (kg/m^3^) was determined by the quotient between the yield and the water applied per season in each treatment.

### Statistical Analysis

Preliminarily, a four-way analysis of variance (ANOVA) considering all the factors (cultivar, season, rootstock, and irrigation) and the double interactions that consider the rootstock factor was performed. This analysis allowed to determine that the cultivar and season factors had a significant effect on most of the variables measured in this study (Supplementary Table 1). Therefore, each season and cultivar were considered separately, like the proposed methodology by Buesa et al. (2021). The variables were analyzed considering a completely randomized design with factorial arrangement, with two factors (rootstock and irrigation) and their interaction (RxI). Variables were subjected to an ANOVA, and the significance of the differences was determined by Tukey's test (*p* ≤ 0.05). Additionally, the percentage of variance explained by each factor (for a given variable) was calculated using the quotient between the sum of squares of the factor and the total, multiplied by 100. On the other hand, boxplots of the main variables were performed. ANOVAs and boxplots were made using the Xlstat Software version 2020.3.1 (Addinsoft SARL, Paris, France).

Regression analyses were performed to establish the relationships between A_n_ vs. g_s_, intrinsic Water Use Efficiency (WUE_i_) vs. g_s_ under both treatments, namely, two cultivars and four rootstocks. For the case of WUE_i_, the data were transformed with the natural logarithm (ln WUE_i_) to increase the linearity of the slope in each cultivar-rootstock regression according to Tortosa et al. (2019).

A meta-analysis was applied to physiological trait responses to deficit irrigation in both cultivars and rootstocks based on Yan et al. (2016) and Zhang et al. (2018). This allowed to determine the different response patterns between cultivars and rootstocks under deficit irrigation, integrating magnitudes of the decline, and integrating results between seasons. The effect size for each observation was calculated as the response ratio (InR) to represent the magnitude of the responses of plant water status to deficit irrigation conditions:


(1) $$InRR=In\left(X_{T1}/X_{T0}\right)=In\;\left(X_{T1}\right)-In\;\left(X_{T0}\right)$$

where *X_T1_* and *X_T0_* are the mean response values of each individual observation in the deficit irrigation treatment and control irrigation conditions, respectively.

The variance of the response ratio (LnR) was calculated as follows:


(3) $$vi=\ln\left[\left(1/n_{T1}\right)\times {\left(S_{T1}/X_{T1}\right)}^{2}+\;\left(1/n_{T0}\right)\times {\left(S_{T0}/X_{T0}\right)}^{2}\right]$$

where n_T1_, n_T0_, S_T1_, S_T0_, *X_T0_*, *X_T1_* and are the sample sizes, standard deviations, and mean response values in the deficit irrigation and control irrigation conditions, respectively. To improve the accuracy of LnR and reduce its variability, the mean weighted response ratio (LnRR++) was calculated from LnRR:


(3) $$\begin{array}{r} lnRR_{++}=\;\overset{m}{\underset{i=1}{\sum }}\overset{k}{\underset{j=1}{\sum }}W_{ij}\;lnRR_{ij}/\overset{m}{\underset{i=1}{\sum }}\overset{k}{\underset{j=1}{\sum }}W_{ij} \end{array}$$

where m is the number of groups (e.g., rootstock), k is the number of comparisons in the *i*th group (measurement number throughout the three seasons), and W is the reciprocal of the variance that was considered as the weight of each LnR and calculated as follows:


(4) $$\begin{array}{r} W=1/vi. \end{array}$$

The meta-analyses were performed using the R software package (version 3.1.1) (R Development Core Team, 2008). The natural logs of the response ratios (RRs) for the individual and combined treatments were determined by specifying the rootstock as a random factor in the model in the “metafor” package. The effects of deficit irrigation on water status and gas exchange were considered significant if the 95% confidence intervals (CIs) of lnRR did not overlap with zero. The bigger the value is, the greater the influence of T1 on the vines. Therefore, to make the lnRR_++_ more visible, it was calculated the percent change (D, %) as follows:


(5) $$\begin{array}{r} D\left(\%\right)=\left(e^{lnRR_{++}}-1\right)\times 100\% \end{array}$$

## Results

### Hyper Arid Conditions Exhibited Reduced Variability Among Seasons

Main climatic characteristics of the three growing seasons under study are shown in Table 1. Vapor pressure deficit (VPD) showed a similar behavior pattern in the three seasons, increasing as the season progresses (from budburst to harvest, mean values). Within the seasons, Season 3 (S3) was the one that presented the lowest VPD value (mean value Bu-Ha, 12% lower) compared to seasons S1 and S2, which were similar (1.0–1.01 kPa). Reference evapotranspiration (ET_0_) showed a similar pattern between seasons, where ~50% of atmospheric demand (ET_0_) occurred in the Flowering-Veraison period (Fl-Ve), with similar values between the different seasons. Growing degree days (GDD) showed a behavior pattern like ET_0_ during the three growing seasons. S3 was the one that exhibited the lowest accumulation of GDD from Budburst to Harvest (Bu-Ha). Precipitation and maximum and minimum temperatures for the three growing seasons are shown in Supplementary Figure 1. The temperature patterns were similar between seasons, with S3 being the one that presented lowest values of minimum and maximum temperatures on average. Rainfall was concentrated during the winter months, with no rain during spring and summer. S2 was a season with lowest rainfall (7.9 mm), while S1 and S3 had more rainfall (36.2 and 52.3 mm, respectively), but far below the historical mean for the study site (96 mm).

**Table 1:** Vapor pressure deficit (VPD), reference evapotranspiration (ET0), and growing degree days (GDD) for the main phenological stages of both cultivars during 2018–2019 (S1), 2019–2020 (S2), and 2020–2021 (S3) seasons.

**Season**	**Variable**		**Bu-Fl**	**Fl-Ve**	**Ve-Ha**	**Bu-Ha**
S1		Min	0.42	0.63	0.65	0.42
	VPD (kPa)	Max	1.65	1.64	1.50	1.65
		Mean	0.96	1.00	1.03	1.00
	ET0 (mm)	Accumulated	186.2	411.8	199.7	797.7
	GDD (°Cd)	Accumulated	308.5	661.4	414.8	1,384.7
S2		Min	0.32	0.55	0.71	0.32
	VPD (kPa)	Max	1.94	1.82	1.52	1.94
		Mean	0.98	1.02	1.03	1.01
	ET0 (mm)	Accumulated	156.8	444.9	190.8	792.4
	GDD (°Cd)	Accumulated	223.8	743.9	387.9	1,355.7
S3		Min	0.39	0.47	0.61	0.39
	VPD (kPa)	Max	1.57	1.27	1.29	1.57
		Mean	0.85	0.87	0.92	0.88
	ET0 (mm)	Accumulated	182.2	405.2	208.2	795.6
	GDD (°Cd)	Accumulated	267.0	636.6	386.1	1,289.7

*Bu, Budburst; Fl, Flowering; Ve, Veraison; Ha, Harvest; Min, Minimum; Max, Maximum*.

### Contrasting Physiological Responses of Cultivars Due to Deficit Irrigation

**Figure 1:**
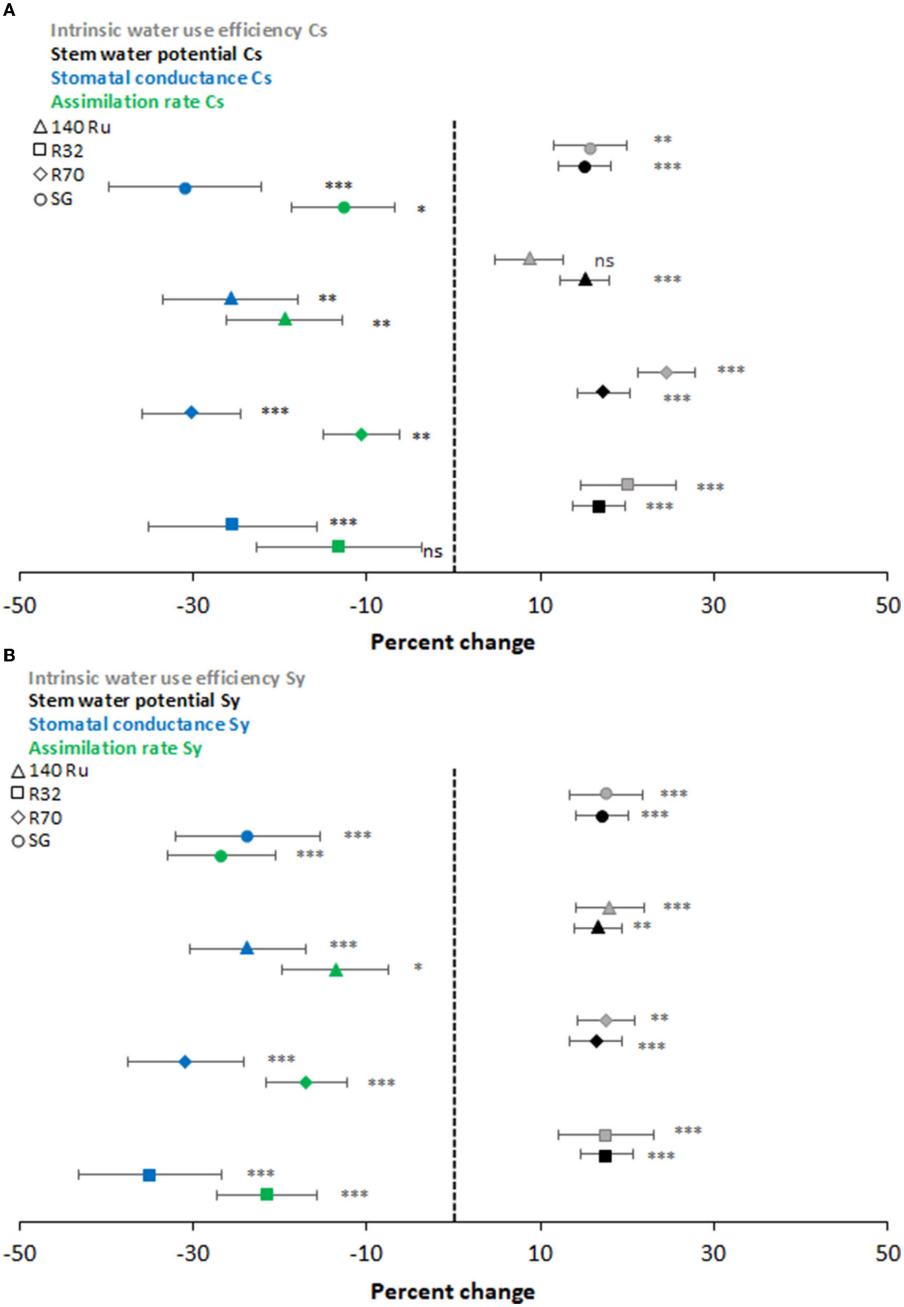
Percent change of intrinsic water-use efficiency (gray), stem water potential (black), stomatal conductance (blue), and assimilation rate (green) under two different moderators: hydric contrasting cultivars (CS—**A**; and Sy—**B**) and rootstocks (140Ru - triangle, R32-square, R70 -diamond, and SG-circle). Asterisks near the symbols specify the significance, and the error bars show the 95% confidence interval (CI).

Overall, the evolution of the gas exchange was rather dynamic throughout the three growing seasons. It only showed significant differences at rootstock level during quite limited days of the season, and mainly observed under deficit irrigation conditions. Under T0 conditions, the A_n_ of CS and Sy on average were 11.1 and 12.3 μmol CO_2_ m^−2^ s^−1^, respectively, and considered all rootstocks (Supplementary Figures 2, 3 for CS and Sy, respectively). Regarding g_s_, both cultivars showed an average of 0.2 mol H_2_O m^−2^s^−1^ under T0 conditions. Plant water status displayed the same seasonal pattern than gas exchange (Supplementary Figures 4, 5 for CS and Sy, respectively). The average Ψ_stem_ during all growing seasons, regardless of the rootstocks, were −0.9 MPa and −1.0 MPa for CS and Sy, respectively (Supplementary Figures 6, 7). In addition, independent of the cultivar and rootstock combination, it was observed that the stress integral (SIΨ) was significantly higher during the last season (−93 MPa), followed by the second (−170 MPa), and finally the first season (−189 MPa). For CS vines, rootstock R70 (−141MPa) reached an SI significantly higher than showed by 140 Ru (−147 MPa) in average, whereas R32 and SG did not differ between both at the end of the study. This SIΨ was similar in Sy vines grafted on the different rootstocks (Table 2). The magnitudes of the T1 effect on physiological parameters during all growing seasons were frequently significant and oscillated to a greater or lesser extent according to the cultivar and rootstock as shown (Figure 1). In the case of CS, T1 decreased the A_n_ of R70, SG, and 140 Ru by 11, 13, and 19%, respectively, while the R32 was not affected. Also, the decrease of g_s_ in the rootstocks R32, 140 Ru, and R70 by T1 were 25, 26, and 30%, respectively, which were lower than the observed in SG that had a decrease of 31%.

**Table 2:** Effect of different rootstocks and irrigation treatments on mean seasonal values of water stress integral (SIΨ).

	**Stress integral (MPa)**			
	**Cabernet Sauvignon**		**Syrah**	
**Rootstock (R)**				
R32	−144	ab	−154	a
R70	−141	a	−153	a
140Ru	−147	b	−161	a
SG	−144	ab	−159	a
**Treatment (T)**				
T0	−133	a	−138	a
T1	−154	B	−174	b
**Season (S)**				
2018–2019	−180	c	−197	c
2019–2020	−160	b	−179	b
2020–2021	−92	a	−94	a
**ANOVA**				
R	0.0551		0.0395	
T	<0.001		<0.001	
S	<0.001		<0.0001	
R × S	0.1758		0.3885	
R × T	0.7049		0.1896	
S × T	<0.0001		<0.0001	

*T0 (control) indicates that vines were irrigated 100% according to actual evapotranspiration. T1 corresponds to a sustained deficit irrigation in which vines were irrigated with the 50% of actual evapotranspiration throughout the experimental period*.

**Figure 2:**
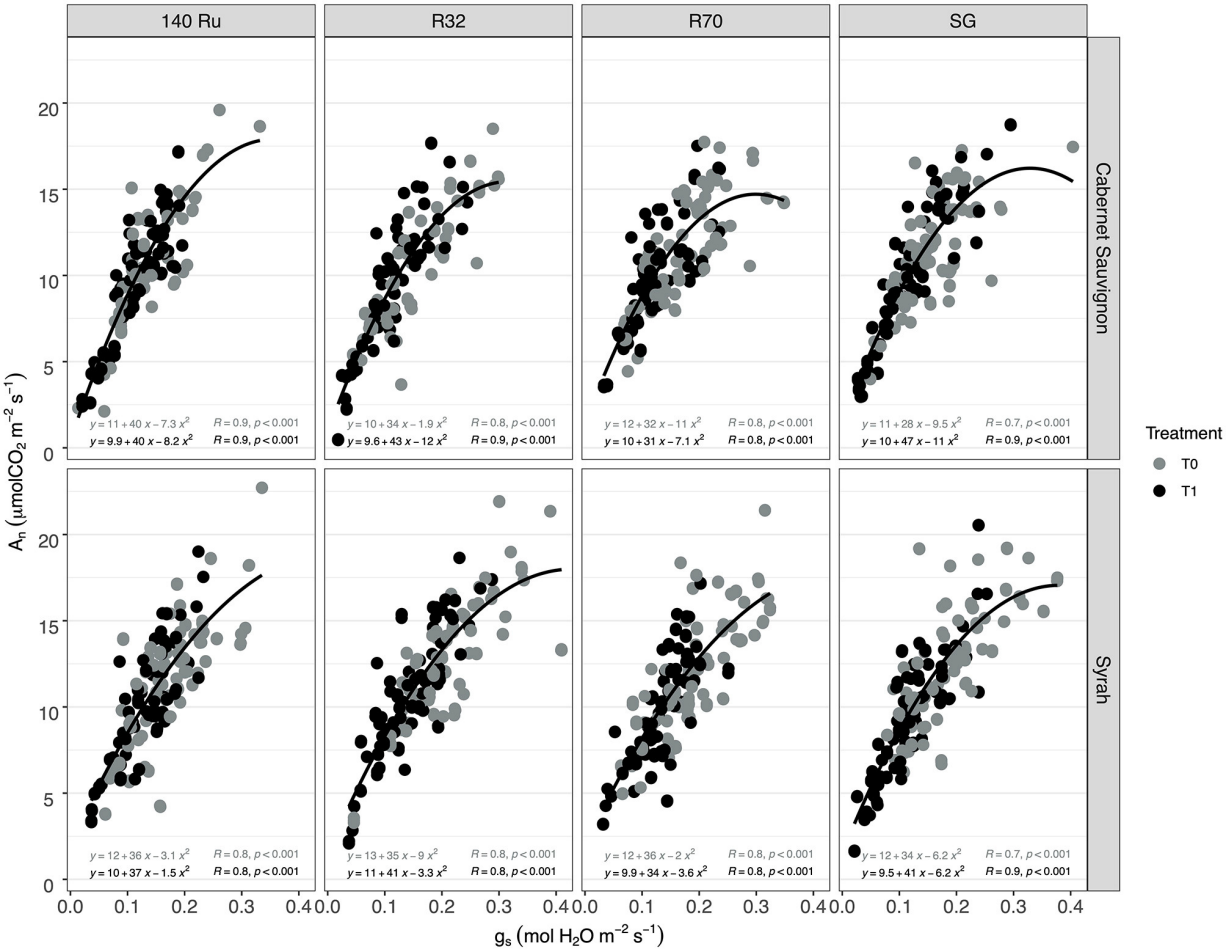
The relationships between photosynthesis (A_n_) vs. stomatal conductance (g_s_) in two cultivars (Cabernet Sauvignon, CS, and Syrah, Sy) over different rootstocks under two irrigation treatments.

Regarding Ψ_stem_, the effect of T1 was significant in all rootstocks, with a decrease between 15 and 17%. The WUE_i_ of CS under T1 was significantly increased by 16, 20, and 25% for SG, R32, and R70, respectively, whereas the effect of T1 was not significant for 140 Ru (9%). Regarding the impact of deficit irrigation (T1) on Sy vines, it was observed that the reductions of A_n_ were significant and that rootstocks 140 Ru (14%), R70 (17%), and R32 (21%) alleviated these reductions. In turn, the reduction of A_n_ in these rootstocks was less than the reduction observed in g_s_. Instead, the reduction of A_n_ in SG was greater than that of the other rootstocks and greater than the reduction in its g_s_. Moreover, the percentage changes in Ψ_stem_ and WUE_i_ induced by T1 were significant and similar for all rootstocks, with a decrease and increase close to 20%. On the other hand, the responses of A_n_ were associated with the variations of g_s_ through a significant non-linear relationship (α = 0.05) under optimal and deficit irrigation conditions for both cultivars and each rootstock as shown in Figure 2. In this regard, with a g_s_ between 0.35 and 0.15 mol H_2_O mol m^−2^ s^−1^, it was observed that the A_n_ of CS and Sy was 12.0 and 13.4 μmol CO_2_ m^−2^ s^−1^ on average. Thus, between g_s_ values of 0.15 and 0.05 mol H_2_O mol m^−2^ s^−1^, these A_n_ values decreased to 8.3 and 7.9 μmol CO_2_ m^−2^ s^−1^ in C S and Sy, respectively. Moreover, in both cultivars, it was observed that the correlation between A_n_ and g_s_ increased with rootstocks under a well irrigated condition (T0). Instead, under deficit irrigation conditions (T1) it was observed that the same correlation decreased with rootstocks (Figure 1).

**Figure 3:**
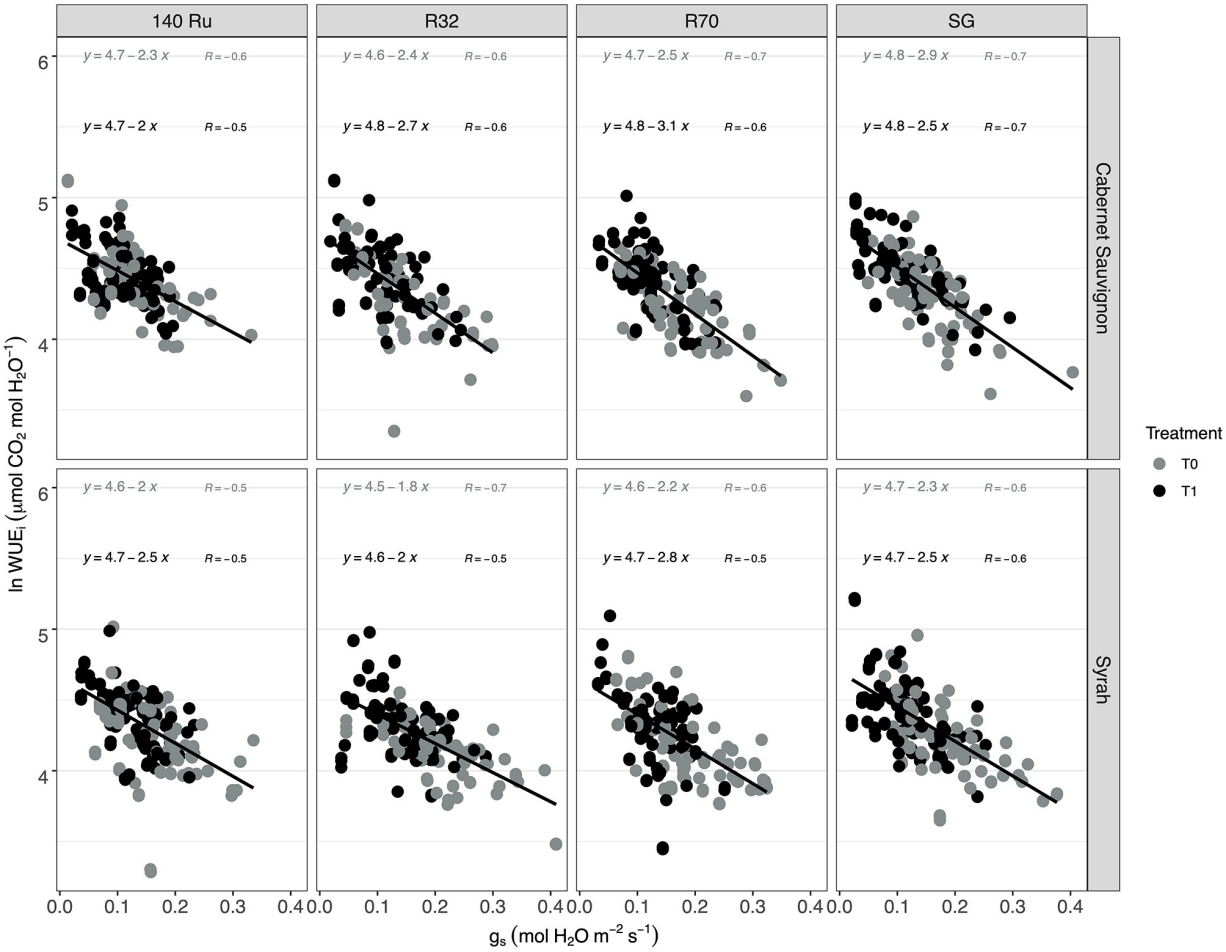
The relationships between linearized intrinsic water use efficiency (ln WUE_i_; A_n_/g_s_) vs. stomatal conductance (g_s_) in two cultivars (CS and Sy) over different rootstocks under two irrigation treatments.

The response of WUE_i_ to the g_s_ variations showed a significant relationship (α = 0.05) under control and deficit irrigation for both cultivars and each rootstock (Supplementary Figure 8). Under mild water stress conditions (g_s_ between 0.15 and 0.35 mol H_2_O m^−2^ s^−1^ as proposed by Medrano et al., 2002), SG and 140 Ru efficiency were 70.5 and 73.1, respectively, whereas the WUE_i_ for R32 and G7 were 67.2 and 64, respectively. Under similar mild water stress conditions, Sy did not shown differences of WUE_i_ between rootstocks with a mean value of 65.6 μmol CO_2_ mol ^−1^ H_2_O. Under higher water stress (g_s_ between 0.05 and 0.15 mol H_2_O m^−2^ s^−1^), CS plants increase their WUE_i_ to 88.7 μmol CO_2_ mol^−1^ H_2_O without differences between rootstocks. For Sy vines in the same treatment, it was observed that the WUE_i_ in R70 was 78.5 μmol CO_2_ mol^−1^ H_2_O, whereas it was 83.3 μmol CO_2_ mol^−1^ H_2_O in average for the other rootstocks. On the other hand, these WUE_i_ responses were linearized by means of natural logarithm. In this regard, the correlation (*r*) under T0 and T1 conditions were higher for self-grafted plants in CS. In turn, Sy vines showed a higher correlation (*r*) in plants grafted onto rootstock R32 and SG under T0 and T1 conditions, respectively. Furthermore, it was observed that SG and R70 displayed a lower slope when they were in T0 and T1 conditions in both cultivars, respectively (Figure 3).

### Irrigation Impacts in Phenology Are Influenced by Rootstocks

**Figure 4:**
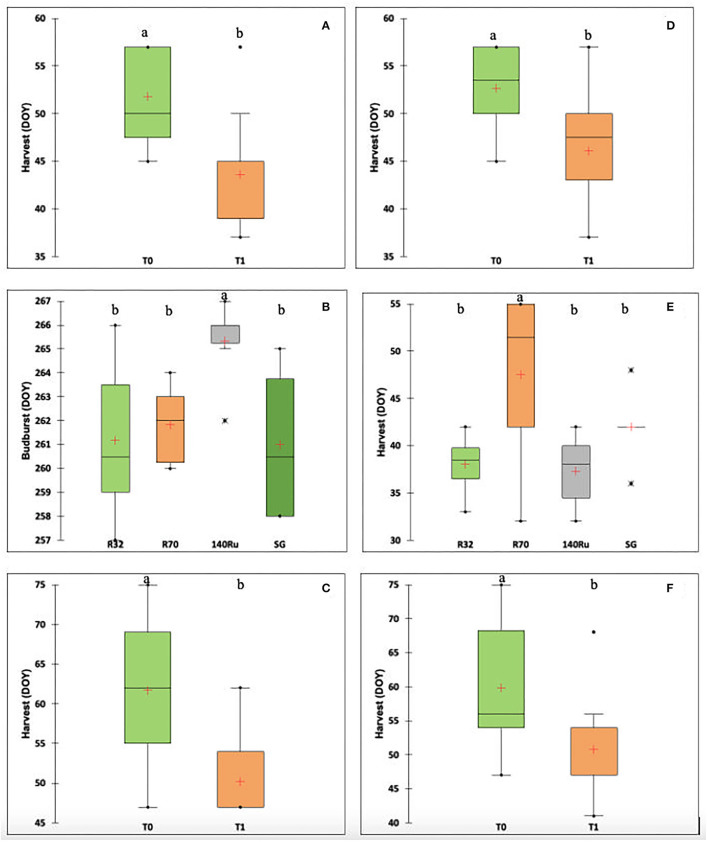
Boxplots showing the distribution of phenological variables by the factor that explained the largest amount of variance for each cultivar and season according to Table 2. **(A)** Harvest (Days of the year, DOY) based on to irrigation, cv CS, 2018–2019 season; **(B)** Budburst (DOY) based on to rootstock, cv CS, 2019–2020 season; **(C)** Harvest (DOY) based on to irrigation, cv CS, 2020–2021 season; **(D)** Harvest (DOY) based on to irrigation, cv Sy, 2018–2019 season **(E)** Harvest (DOY) based on to rootstock, cv Sy, 2019–2020 season; and **(F)** Harvest (DOY) based on to irrigation, cv Sy, 2020–2021 season. Within each figure, different lowercase letters present significant differences (*p*-value < 0.05).

The percentage of variance explained by rootstocks, irrigation, and interaction for the phenological variables according to cultivar and season are shown in Table 3 (only the significant ones). As mentioned, in a more complete exploratory analysis, the factor that explained the greatest variance for the phenological variables corresponded to the season. This in turn considered the effect of the season, the cultivar, and their interactions (Supplementary Table 1). Regarding the rootstock factor, for both cultivars, there were specific effects on the date of occurrence of the main phenological stages during the 2019–2020 season. The irrigation factor affected the harvest date in both cultivars in two of the three seasons under study. For the interaction (RxI), there were specific effects in both cultivars. For the S1 and S3 seasons, the harvest date in both cultivars was earlier (between 8 and 10 days) in the irrigation treatment T1 (Figures 4A,C,D,F). For CS, the budburst date (S2) was affected by the different rootstocks, where 140-Ru presented a later budburst date (4 days later) compared to the other rootstocks (Figure 4B). For Sy, during the S2 season, the harvest date was affected by the rootstocks, where R70 presented a later harvest date (8 days later) compared to the other rootstocks (Figure 4E).

**Table 3:** Percent of variance explained by each factor (Rootstock and Irrigation) and the interaction (RxI) for the phenological variables in each cultivar and season.

**Season**	**Variable**	**Cabernet Sauvignon**			**Syrah**		
		**R**	**I**	**RxI**	**R**	**I**	**RxI**
S1	Budburst						
	Flowering						
	Veraison						
	Days Bu-Ve			29.2			
	Harvest (22.5°Brix)		36			27	
S2	Budburst	33.9					30.3
	Flowering						
	Veraison						
	Days Bu-Ve						
	Harvest (22.5°Brix)				37.2		
S3	Budburst						
	Flowering						
	Veraison						
	Days Bu-Ve						
	Harvest (22.5°Brix)		37.1			27	

*Only factors which explained a significant portion of the variance (p < 0.05) are plotted. The percent variance explained by each factor is indicated using the value (%) and color. Darker colors explain a higher variance in each cultivar and season*.

*R, Rootstock; I, Irrigation; S1, 2018–2019 season; S2, 2019–2020 season; S3, 2020–2021 season; Days Bu-Ve, Days between budburst and veraison*.

**Figure 5:**
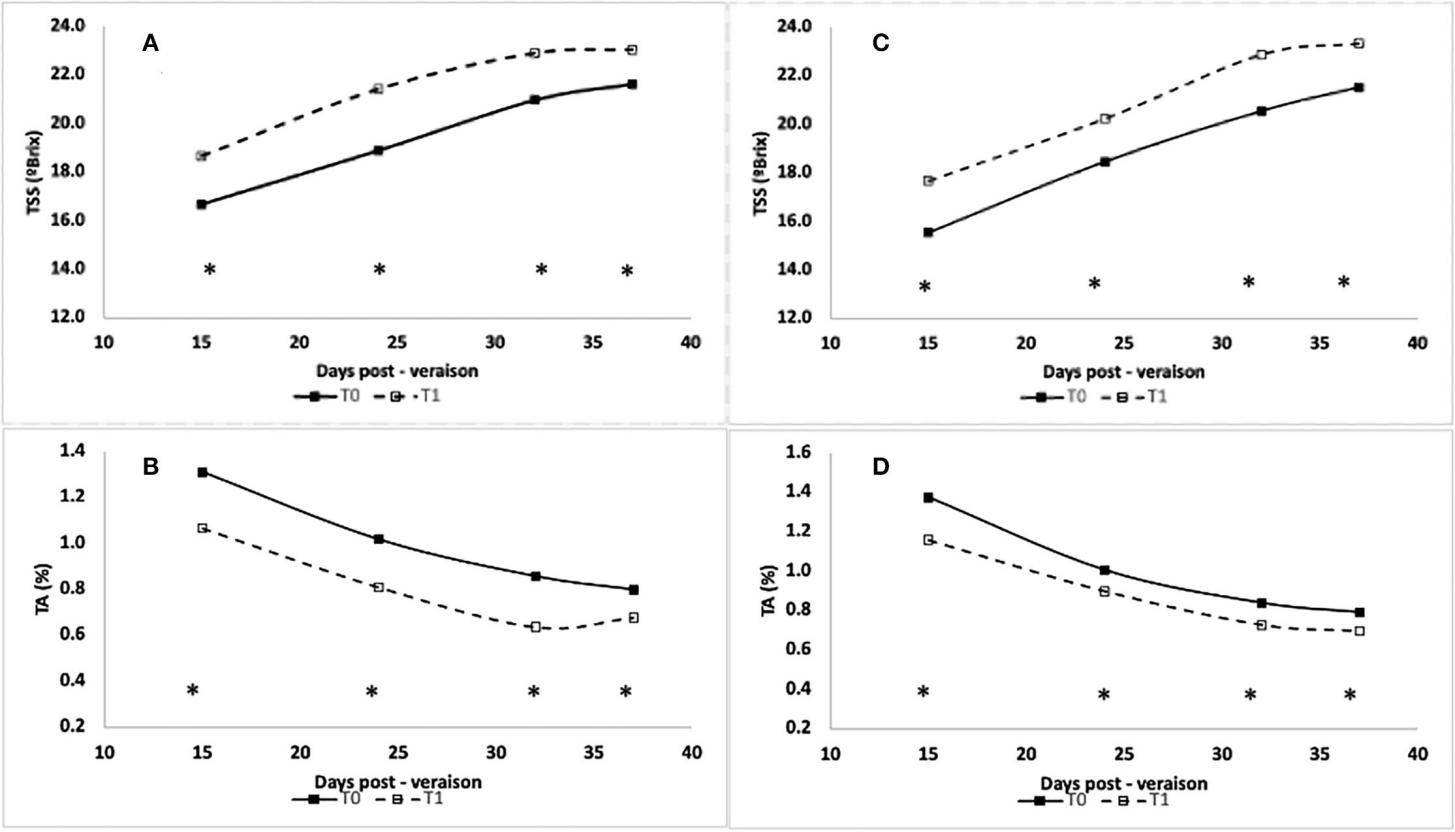
Total soluble solids (TSS, °Brix, **A,C**) and Titratable Acidity (TA, %, **B,D**) measured at different days post—veraison for CS (**A,B**) and Sy (**C,D**) cultivars during the 2018-2019 season (S1). Asterisk (*) represents significant differences (*p*-value < 0.05).

The percentage of variance explained by rootstocks, irrigation, and interaction for the berry maturity evolution, according to cultivar and season (only the significant ones), is shown in Table 4. Rootstock factor affected the evolution of berry maturity, particularly for some dates and seasons, in both cultivars without consistency. On the other hand, the irrigation factor affected the three maturity parameters considered in almost all the dates of the 2018–2019 season (S1) for both cultivars, being more consistent for total soluble solids (TSS) and titratable acidity (TA). For the other seasons, the effect of the irrigation factor was not consistent, with specific effects in some maturity parameters and specific dates in both cultivars (Table 4). Regarding the interaction (RxI), it only affected some parameters and specific dates. During the 2018–2019 season (S1), treatment T1 (irrigation) presented for all sampling dates higher values of total soluble solids in both cultivars (Figures 5A,C) and lower values of titratable acidity (Figures 5B,D) with respect to the control (T0). The differences in total soluble solids were ~2°Brix, independent of the cultivar and sampling date, while for titratable acidity, the differences were higher in the CS cultivar (0.2%) than in Sy (0.13%).

**Table 4:** Percent of variance explained by each factor (Rootstock and Irrigation) and the interaction (RxI) for the maturity variables in each cultivar and season.

**Season**	**Days post-Veraison**	**Variable**	**Cabernet Sauvignon**			**Syrah**		
			**R**	**I**	**RxI**	**R**	**I**	**RxI**
S1	15	TSS	26.9	33.2			30	
		TA	22.9	41.1			47.6	
		pH						
	24	TSS		56			21.4	
		TA		53.4			24.8	
		pH		25.2				
	32	TSS		35.4			34.7	
		TA		74.7			52.4	
		pH		36.1				
	37	TSS		32.1			26.5	
		TA		43.7			45.7	
		pH						
S2	5	TSS					14.6	
		TA						
		pH						47
	12	TSS					16.7	
		TA						
		pH						
	24	TSS				38.3	11.7	
		TA		23.8		44		
		pH		26.7		37.9		
	32	TSS						
		TA	35.5			42.2	13.6	
		pH	41.2					
S3	4	TSS						
		TA						
		pH						
	12	TSS						
		TA					21.6	
		pH						
	22	TSS						
		TA		21.9	35.1		17.8	
		pH						
	30	TSS		21.9			28.9	
		TA		23	35.5		50.3	
		pH						

*Only factors which explained a significant portion of the variance (p < 0.05) are plotted. The percent variance explained by each factor is indicated using the value (%) and color. Darker colors explain a higher variance in each cultivar and season*.

*R, Rootstock; I, Irrigation; S1, 2018–2019 season; S2, 2019–2020 season; S3, 2020–2021 season; TSS, Total soluble solids; TA, Titratable Acidity*.

### Productivity Traits Are Modified by Treatment and Influenced by Both Cultivars and Rootstocks

Considering the season effect and its impact as a large source of variation captured in the measurements and experimental set-up and expected influence by cultivar scion (Supplementary Table 1; Table 5), we also measured the percentage variance in traits explained by rootstocks, irrigation, and their interaction (RxI). The highest variance percentage explained among Rootstock by Irrigation interaction was Pruning Weight trait in both cultivars CS and Sy in two out of three seasons. Particularly, in Season 1 where pruning weight trait were 28.6 and 23.5%, and in Season 3 were 34.1 and 18.5%. In season two, the most significant interaction RxI was the Caliber trait for CS (35.8%), followed by Rachis Length (31.8%) and Bunch number per plant (29.7%) in this cv. No significant interactions in RxI were detected in Sy during Season 2. Irrigation treatments also had a significative impact in several traits during the three seasons considered. The highest variance percentage explained during the first season was Pruning Weight (29.7%), followed by Ravaz Index (18.7%) in CS, Meanwhile, in Sy, the traits displayed a different composition, with Water Productivity with the highest variance explained (44.5%), followed by Yield (36.5%). During the second season, Ravaz Index displayed a higher variance (28.8%), followed closely by Water Productivity (27.7%) in CS vines. Again, during in this season, the highest variance percentages in Sy vines were exhibited by Trunk Circumference (31.1%) and Yield (30.5%). Finally, in the third season, Water Productivity (15.8%) and Trunk Circumference (10.6%) displayed the highest variance in CS vines. In Sy vines, Trunk Circumference (43.9%) displayed the highest variance, followed by Ravaz Index (37.2%). Interestingly, the variance percentages were higher in Sy in response to irrigation treatments.

**Table 5:** Percent of variance explained by each factor (Rootstock and Irrigation) and the interaction (RxI) for the productive variables in each cultivar and season.

**Season**	**Trait**	**Cabernet Sauvignon**			**Syrah**		
		**R**	**I**	**RxI**	**R**	**I**	**RxI**
**S1**	Yield	35.1	18.5			36.5	
	N° Bunch per plant	43.8					
	Bunch Weight					33.4	
	N° Berries per bunch	-	-	-	-	-	-
	Berry weight	-	-	-	-	-	-
	Rachis length	-	-	-	-	-	-
	Rachis weight	-	-	-	-	-	-
	Caliber	-	-	-	-	-	-
	Water productivity	28.8	16.2			44.5	
	Pruning weight	37.3	29.7	28.6	21.1	27.2	23.5
	Trunk circumference	68.7		24.4	27.8	21	
	Ravaz index	35.6	18.7				
**S2**	Yield	57.7		29.9		30.5	
	N° Bunch per plant	40.3	10.6	29.7			
	Bunch weight					28.4	
	N° Berries per bunch						
	Berry weight						
	Rachis length			31.8			
	Rachis weight					22.9	
	Caliber			35.8		14.3	
	Water productivity	31.9	27.7				
	Pruning weight	38.6					
	Trunk Circumference	69.2	6	19.7		31.1	
	Ravaz index		28.8				
**S3**	Yield	50			29		
	N° Bunch per plant						
	Bunch weight						
	N° Berries per bunch						
	Berry weight				28.7	32.1	
	Rachis length						
	Rachis weight						
	Caliber				25.9	33.6	16.7
	Water productivity	32.9	15.8			20.3	
	Pruning weight	56.1	7.4	34.1	23.2	28.2	18.5
	Trunk circumference	68.4	10.6	17.2		43.9	
	Ravaz index					37.2	

*Only factors which explained a significant portion of the variance (p < 0.05) are plotted, and percentage of variance explained is indicated using the value (%) and color. Darker colors explain a higher variance in each cultivar and season*.

*R, Rootstock; I, Irrigation; S1, 2018–2019 season; S2, 2019–2020 season; S3, 2020–2021 season. - data not available*.

Rootstock explained a significant amount of variation in most traits measured in CS vines, in contrast to the behavior of Sy that explained most traits in response to Irrigation treatment. As such, the strongest effect for Trunk Circumference (68.7%), Bunch Number per plant (43.8%), Pruning Weight (37.3%), Ravaz Index (35.6%), Yield (35.1%), and Water Productivity (28.8%) were observed in CS vines in Season 1, while only Trunk Circumference (27.8%) and Pruning Weight (21.1%) were significant in Sy vines. During second season, the traits Trunk Circumference (69.2%), Bunch Number per plant (40.3%), Pruning Weight (38.6%), and Water Productivity (31.9%) displayed the strongest effect for CS. No significative effects were determined for Sy during this season. The third season recorded the strongest effect for Trunk Circumference (68.4%), followed by Pruning Weight (56.1%), Yield (50%), and Water Productivity (32.9%) in CS. In turn, Syr effects were significant in Yield (29%), Berry Weight (28.7%), Caliber (25.9%), and Pruning Weight (23.2%) for Season 3.

### Rootstock Performance in Deficit Irrigation Is Linked to Cultivar Behavior

**Figure 6:**
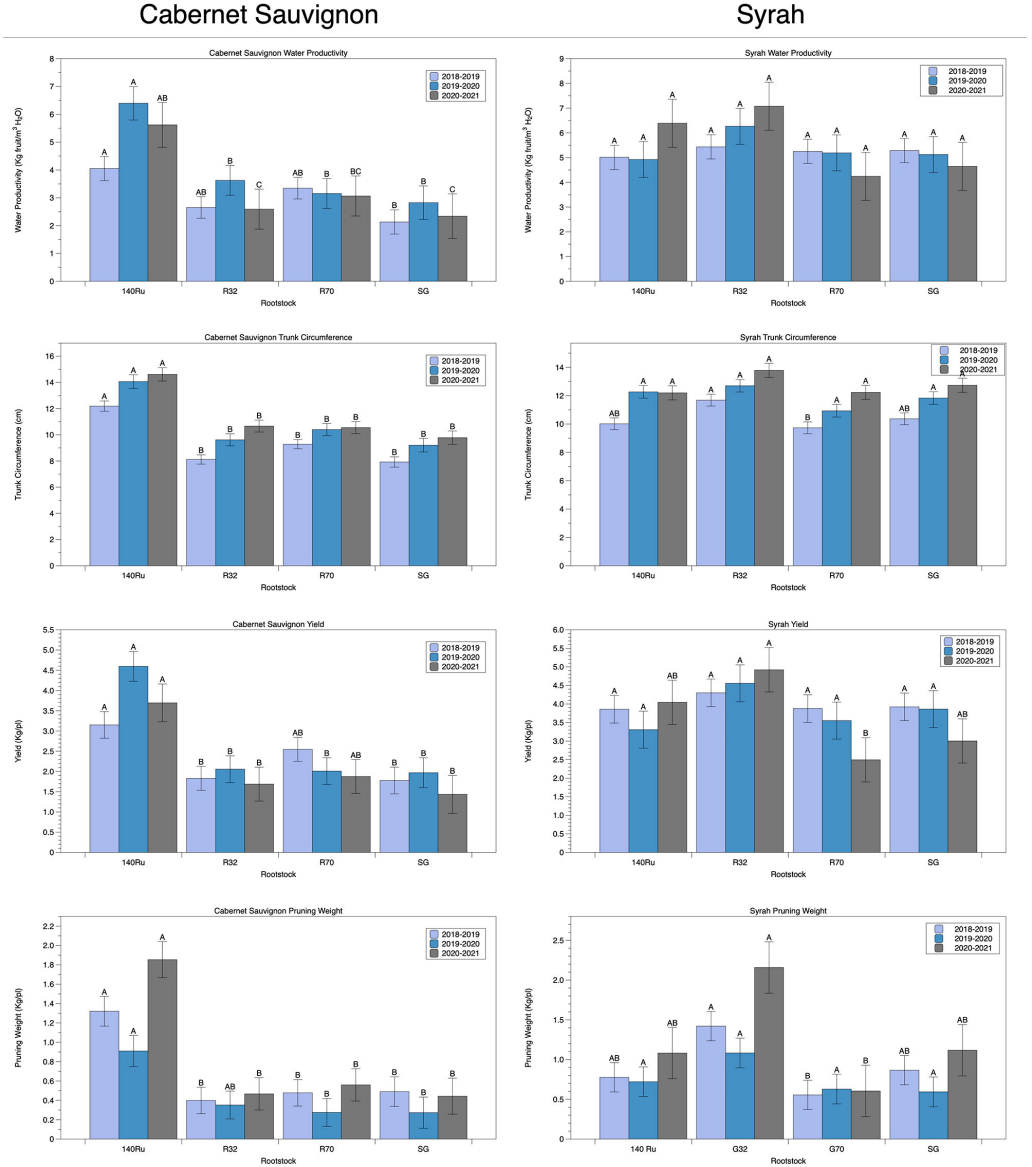
Functional responses of selected rootstocks for productivity attributes, including Water Productivity, Trunk Circumference, Yield, and Pruning Weight among seasons and cultivars. Within each panel, different letters represent significant differences (*p*-value < 0.05).

Focusing on the traits in which rootstock showed the strongest effect, we plotted the distributions for Water Productivity, Trunk Circumference, Pruning Weight, and Yield among all seasons (Table 5) and compared each of the rootstocks using a Tukey test (Figure 6). A common trend was observed, which was determined by the interaction with the cultivar scion. Commercial rootstock 140Ru obtained higher results and surpassed R32, R70 and SG (grafted control) in CS vines. Conversely, R32 exceeded the rest of rootstocks considered in the analyses, exhibiting the highest water productivity, trunk circumference, yields and pruning weights in Sy vines, although without significance in WP (but with decreases ranging from 13% to 22%) as shown in Figure 6. Trunk circumference in Sy was significantly higher for R32 (12.7 cm average with reductions of 10, 14, and 9% in 140 Ru, R70, and SG, respectively). The average yield for R32 in Sy (4.6 Kg/plant) was significantly higher than the other rootstocks and grafting control evaluated (a comparative decrease of 19, 28, and 22% for 140Ru, R70, and SG, respectively). Another productive trait was pruning weight, where R32 significantly exceeded the other rootstocks tested and controlled by having an average of 1.6 Kg/pl in three seasons compared to other tested rootstocks (0.9, 0.6, and 0.9 Kg/pl in 140 Ru, R70, and SG, respectively) (Figure 6). Similar results were observed when considering the behavior of the rootstocks in both cultivars under the T0 treatment (data not shown).

## Discussion

This study was designed to determine to what extent the plant water relations are modified by using naturalized rootstocks and whether this fit primary productivity and adaptation to harsh conditions typical of a hyper-arid zone that is expected to occur in Mediterranean regions due to CC (Morales-Castilla et al., 2019; IPCC, 2021). Considering the observed decline of precipitation over central Chile, which has been greatly accentuated by an uninterrupted sequence of dry years from 2010 to the present, with annual rainfall deficits ranging between 25 and 45% (Garreaud et al., 2020), the 50% deficit irrigation was a feasible projected decrease. This ongoing, multiyear dry spell has been referred to as the Central Chile Mega Drought (MD) due to its unprecedented longevity and large spatial extent in the historical record (CR2, 2015). Nevertheless, the phenological, physiological, and productive responses necessarily respond to the climatic conditions that are provisional between the growing seasons. Within the productive variables, grapevine phenology is fundamental for the planning of agricultural practice within the fields. For example, irrigation, fertilization, and the application of phytosanitary and hormonal products are programmed based on the date of occurrence of phenological states such as budburst, flowering, fruit set, and veraison. Indeed, the main factor that affects grapevine phenology is related to temperature (Parker et al., 2011). In this sense, the “season” factor, related to different climatic conditions (Table 1), was the most significant for the different phenological stages (Supplementary Table 1). The growing degree days accumulation (between budburst and harvest) did not vary between seasons (1,300–1,400 heat units), being like those reported in other wine-growing zones of Chile for the cv CS (Verdugo-Vásquez et al., 2016). Regarding the effect of the use of rootstocks and irrigation on the grapevine phenology, there is little information in literature (Keller et al., 2012; Sabbatini and Howell, 2013). It has been reported that modifications in the date of occurrence due to the use of rootstocks are related to indirect scion response, such as canopy density, as influenced by the rootstock's direct impact on scion vigor (Sabbatini and Howell, 2013). In this study, there were specific effects at the beginning of the season (budburst) for the cv CS (differences <5 days, Season S2), but the differences observed at the beginning of the season were not maintained throughout the season without significant differences for flowering, veraison, and harvest. For cv Sy, the differences due to the use of rootstocks were at the end of the season (harvest), but were also not consistent between seasons. It was observed that there is no consistency in the results between cultivars and seasons, which is why long-term studies are required to determine whether the use of rootstocks allows advancing or delaying phenological stages stably and the mechanisms by which differences are generated. For the “irrigation” factor, there were more consistent results between cultivars and seasons, wherein the decrease in irrigation (T1) advanced harvest date (at the same level of total soluble solids) for both cultivars. This advance was related to the accumulation of sugars, rather than differences in the beginning of the ripening period (veraison) since there were no differences due to the irrigation factor for budburst, flowering and veraison. At the within-field scale, it was determined that soil conditions (slope and total soil water availability) can affect grapevine phenology at the beginning of the season (budburst), and is associated with differences in the initial soil moisture (Li et al., 2016; Verdugo-Vásquez et al., 2021b). However, in this study, despite the differences in the water applied since the beginning of the season and the absence of rain in winter, there were no significant differences due to the irrigation factor for budburst.

Regarding fruit maturity, the “rootstock” factor only modified some specific dates and was not consistent between seasons. Similar results were observed by Keller et al. (2012), as they reported that scion effects and differences due to yearly climate variation far outweighed any differences due to rootstock for fruit maturity. On the other hand, the “irrigation” factor was more consistent in the results, showing that the decrease in irrigation increases the accumulation of sugars in the berries and decreases the titratable acidity, being the earliest harvest, as mentioned above. These results coincide with those reported in literature (Acevedo-Opazo et al., 2010; Cabral et al., 2021; Pérez-Álvarez et al., 2021), according to the time and intensity of water stress (Romero et al., 2022). The differences observed in maturity were more related to the general balance of grapevine (Ravaz index) than to berry weight in this study (Table 5).

Previous studies have also experimented with significantly different seasons, which predominate over the physiological parameters, such as Ψ_stem_ and gas exchange, that present dynamic fluctuations itself (Bascuñán-Godoy et al., 2017; Buesa et al., 2017; Romero et al., 2018; Levin et al., 2020). However, there is a high degree of co-regulation in the plant to cope with water deficits through their stomata. Since stomatal closure and regulation of leaf gas exchanges with the atmosphere, it is a key process in response to moderate water deficits both in the soil and in the atmosphere, therefore integrating internal signaling and environmental cues and complex genetic control and thus providing multiple layers of regulation to balance water loss and CO_2_ assimilation in dry environments (Chaves et al., 2016; Levin et al., 2020). In a physiological framework, several studies have already reported the different degrees of responses between cultivars, of which CS and Sy display contrast hydric strategy (Hochberg et al., 2012; Franck et al., 2020; Levin et al., 2020). In this fashion, the meta-analysis showed the extent of reductions in physiological parameters measured at T1 in contrast to T0. Thus, when integrating the effect magnitude of water deficit in CS self-grafted plants, it was determined that the decline of g_s_ was greater than that of the A_n_. On the contrary, in Sy self-grafted plants, the decline magnitude was greater in A_n_ than in g_s_ (Figure 1). On the other hand, the rootstocks alleviated the percentual reductions of A_n_ caused by T1, which agreed with what was reported by Franck et al. (2020), who observed that the naturalized rootstocks grown in pots and under field conditions displayed a better performance.

During water deficit, photosynthesis is limited by both stomatal closure and impairment of the photosynthetic machinery (i.e., metabolic factors) in order to prevent dehydration, during which root system is the major interface between the plant and water availability of a drying soil (Gambetta et al., 2020). Thus, the rootstocks can contribute to the scion water loss through a combination of hydraulic and hormonal root-to-shoot signaling (Lovisolo et al., 2010). In this sense, the relationship of A_n_ vs. g_s_ observed in two contrasting cultivars suggested that different rootstock genotypes (140 Ru, R32, R70) changed the A_n_ sensitivity to g_s_ variations given the coefficients r obtained (Figure 2). Moreover, in the case of CS, the g_s_ values (mol H_2_O m^2^ s^−1^) in which the A_n_ reaches the “Plateau” increased with the use of rootstocks (0.17 SG <0.19 R32 <0.22 R70 <0.26 140 Ru). Meanwhile, Sy decreased with R70 (0.23 mol H_2_O m^2^ s^−1^), remained with 140 Ru (0.26 mol H_2_O m^2^ s^−1^), and increased with R32 (0.29 mol H_2_O m^2^ s^−1^), suggesting a modulation effect. Considering A_n_ and g_s_ behavior, and from an efficiency perspective (WUE_i_), rootstock may also modulate the variability in response to stomatal closure (correlation r), where calculated slopes demonstrated that 140 Ru and R32 were the most conservative for CS and Sy, respectively, in relation to WUE_i_—g_s_ through regressions lines, enabling to compare the slopes between genotypes to highlight environmental and genetic differences (Tortosa et al., 2019).

It has been described that the regulation of stomatal closure is mediated by hydraulic, chemical, physical, and even electrical signals (Beis and Patakas, 2010). Among these signals, an integrated modeling approach suggested that both hydraulic and chemical signals are likely important for the rootstock-specific stomatal regulation. In addition, the coupled chemical-hydraulic factors most precisely describe the stomatal conductance underlying gas exchange of grafted grapevines, since factors controlling ABA biosynthesis (either in leaves or roots, or root system architecture) caused differences in the hydraulic conductance between the rhizosphere and the soil–root interface (Peccoux et al., 2018). In this sense, this study provides results that reinforces the previous conclusions obtained in which the hydraulic variability between grape cultivars is in turn strongly influenced by the ambient VPD (Villalobos-González et al., 2019; Gambetta et al., 2020). In the face of duration and intensity of water stress given by SIΨ, CS was sensitive to the accumulated effects of deficit irrigation at the end of the growing seasons. A naturalized rootstock R32 showed the least stress, even surpassing 140 Ru, which has been shown to be drought tolerant (Romero et al., 2018). Meanwhile, the SIΨ of Sy was indifferent to deficit irrigation in much of the experimental period. The SIΨ values agrees with what was reported by Zúñiga et al. (2018). However, the stress levels in this study were higher than those previously reported and are explained by the conditions of greater aridity in the experimental site as a natural laboratory for CC adaptation studies.

Nowadays, water use (i.e., the water consumed) and WUE (i.e., the efficiency of this consumed water to assimilate carbon, produce biomass, or fruit yield) are crucial parameters, especially in areas with increasing water scarcity that requires adaptation to CC, such as south Europe, West Asia, Western Australia, Chile, North Africa, and parts of South Africa (FAO, 2018; van Leeuwen et al., 2019; Naulleau et al., 2021). Despite the importance of rootstocks for the total water productivity and WUE of the crop, the variability of WUE in rootstocks has largely been underexplored, in addition to obtaining contradictory results (reviewed by Medrano et al., 2018). Moreover, the main role of rootstocks in plant water economy leads to the consideration of the genetic variability of WUE as a complementary target for current research (Medrano et al., 2018). In this regard, differences based upon the interaction among cultivar scion: commercial rootstock support the necessity of exploring differences both in cultivar hydric behavior and the interaction with the rootstocks, leading to change in crop performances depending on the cultivar (Tortosa et al., 2019; Franck et al., 2020; Romero et al., 2022). Thus, a tolerant rootstock, such as 140 Ru, was superior in the hydric conservative CS vines, whereas R32 overcame the 140 Ru performance in Sy vines, which were measured in traits such as water productivity, trunk circumference, and yields and pruning weights (Figure 6). Latter results may indicate enhanced abilities for water uptake and assimilation since near-anisohydric cultivars bears a “risky strategy” of water use. Indeed, 140 Ru exhibited better adaptive behavior as CS vines maintained slight unchanged A_n_, g_s_, and WUE_i_ levels under both well-watered and persistent water stress conditions. This might be associated to greater root water-uptake capacity and whole-plant hydraulic conductance, translating to high productivity and vigor (Romero and García-García, 2020; Romero et al., 2022). With respect to normal rainfall scenarios which could represent the T0 irrigation treatment (100% ETa applied), it was observed that rootstocks behavior differed in each cultivar under these conditions. For cv CS, 140 Ru was the one that presented the best behavior regarding the productive variables analyzed (N° bunches per plant, pruning weight, trunk circumference, among others), while R32 was the one that presented the best behavior with respect to the productive variables analyzed for cv Sy (yield, trunk circumference, water productivity, among others). These results highlight the need to carry out studies that consider different rootstock-scion combinations under different edaphoclimatic conditions.

In concert, CS displayed a conservative behavior in terms of lower g_s_ and high rates of A_n_-enhanced WUE_i_ (Zamorano et al., 2021). Conversely, cv Sy showed increased WUE_i_ due to reduced g_s_ near-anisohydric behavior. This was characterized by less prone to rapid stomatal closure under water stress maintaining higher stomatal aperture and exhibiting substantial reductions in Ψ_stem_. Thus, adjusting locally with lower stomata sensitivity to drought-induced ABA and being more dependent on hydraulic signals such as leaf water status (Coupel-Ledru et al., 2017). In this regard, the integral water stress observed in this study highlighted this condition since cv Sy was the one that accumulated more stress. This cultivar is efficient in terms of water use but shows limited heat dissipation due to its reduced g_s_, which can favor the occurrence of leaf sunburn under severe heat stress and drought (Costa et al., 2012). Other authors have demonstrated the mechanisms by which rootstocks modify the mentioned productive variables and found that the grafted plant modifies the absorption of light, increasing the assimilation of carbon compounds and therefore increasing the yield (Corso et al., 2016; Bascuñán-Godoy et al., 2017).

Recently, a signaling communication peptide has been identified in response to drought, where CLE25 peptide is produced in the roots and moves systemically through the plant vasculature to leaves to drive ABA production by activating biosynthetic enzyme NCED3 (Takahashi et al., 2018). This burst of ABA synthesis leads to stomatal closure and improved water balance, thereby promoting drought survival. This insight into small-peptide signaling in Arabidopsis may help to unravel conserved mechanisms in crops for root-to-shoot mobilization of stress signals (Gupta et al., 2020). Hence, the interaction with the rootstock and the ability of this to later explore and uptake water is fundamental for efficiently facing water deficit. Many traits and mechanisms are involved in the response of a rootstock by scion combination to water demand/water availability ratio. Hence, determining the optimal combination may enhance this adaptative processes. Rootstocks can differ by their capacity to extract water from the soil, which is primary linked to root biomass and to the hydraulic conductivity of the roots (Gomès et al., 2021). Lately, adaptation of viticulture requires a proper exploration of an optimal interaction of cultivar and rootstock, particularly when exploring new geographical areas, new training systems, new management practices, or new varieties, both for rootstocks and scions (Gomès et al., 2021). There are additional studies of water productivity in vineyards, but knowledge of the effect of irrigation reductions and its combined effect with grafted vines using rootstocks on secondary metabolism is still growing (Cáceres-Mella et al., 2018).

Hence, adaptations to CC require modifications in genotypes and viticulture techniques that can influence both phenology expression and grape ripening since rootstocks are able to trigger transcriptional changes on berry secondary metabolism which is relevant for berry composition and sensory properties (Berdeja et al., 2015). Moreover, studies for rootstocks conferring higher drought tolerance to the scion, driving carbon flux toward both accumulation of phenolic compounds, and alteration of anthocyanin profile, thereby altering grape quality at harvest, might be a target (Zombardo et al., 2020). In this regard, rootstocks that have novel drought tolerance mechanisms (i.e M4 rootstock) have shown greater synthesis of phenolic compounds such as stilbenes and flavonoids with enhanced capacity to scavenge and regulate the reactive oxygen species (ROS) levels that are generated under stress conditions and cause oxidative damage (Corso et al., 2015). In the naturalized R32 rootstock tissues grafted to cv Sy, the upregulation of genes of Phenylpropanoid metabolic process and pigment accumulation was determined in response to water deficit (Franck et al., 2020), enhancing plant survival in the presence of abiotic stress. M-rootstocks have also displayed adaptive traits, such as reducing the stomatal conductance and stem water potential while maintaining high photosynthetic activity with high Water Use Efficiency in water-limiting conditions (Bianchi et al., 2020). The capacity of M4 to satisfy the water demand of the scion under limited water availability has shown a delayed stomatal closure, allowing higher photosynthetic activity, which is also related to a reduced activation of ABA signaling both in the root and the leaf level (Prinsi et al., 2021). Therefore, the use of drought tolerant genotypes (scion, clones, and rootstocks) represents an environmentally friendly and cost-effective tool for adaptation to a changing climate (van Leeuwen et al., 2019).

## Conclusions

Rootstock did not modify the main phenological stages, while irrigation treatment allowed modifying the harvest date. Moreover, harvest date and acidity were modified by deficit irrigation treatment and rootstocks did not modulate phenological stages. Adaptation of grapevines to expected lower water availability might be improved by using suitable tolerant rootstocks. In addition, maturity index can be modified through irrigation management.

The regulation and behavior of several physiological parameters related to the plant water status were contrasting among the cultivars studied. In turn, this behavior varied depending on how stressful the environmental conditions were between growing seasons. Under water deficit conditions, when photosynthesis was mainly limited by stomatal conductance, rootstocks showed the ability to adjust the sensitivity by which photosynthesis was restricted.

The dynamic responses and grapevine adaptation to water deficit were highly dependent on the cultivar hydric strategy (near-isohydric or near-anisohydric) and the interaction with the rootstock. Hence, the vine fitness performance will be determined by new environmental demands that will be imposed by climatic challenges, and such growth and developmental responses to drought, higher temperatures, or combined abiotic stresses will rely on the proper combination of both cultivar and rootstock.

## Data Availability Statement

The raw data supporting the conclusions of this article will be made available by the authors, without undue reservation.

## Author Contributions

AZ-S conceived and designed the experiments. EV-S conduced the physiological analyses and laboratory determinations with the guidance of AZ-S, NV-V, and ID. AZ-S wrote the manuscript with the help of EV-S, NV-V, and ID. EV-S, NV-V, and AZ-S prepared all the figures. All authors contributed to the discussion of ideas, revised, and approved the final manuscript.

## Funding

This work was supported by Consejo Nacional de Ciencia y Tecnología CONICYT–Fondecyt Regular (Grant No. 1140039 2014/INIA) to AZ-S, and by Agencia Nacional de Investigación y Desarrollo ANID—Postdoctoral Fondecyt (Grant No. 3180252 2018/INIA) to NV-V.

## Conflict of Interest

The authors declare that the research was conducted in the absence of any commercial or financial relationships that could be construed as a potential conflict of interest.

## Publisher's Note

All claims expressed in this article are solely those of the authors and do not necessarily represent those of their affiliated organizations, or those of the publisher, the editors and the reviewers. Any product that may be evaluated in this article, or claim that may be made by its manufacturer, is not guaranteed or endorsed by the publisher.
