# Supplementary material for: Adapting Grapevine Productivity and Fitness to Water Deficit by Means of Naturalized Rootstocks
Source: Front Plant Sci. 2022 May 24;13:870438. doi: 10.3389/fpls.2022.870438 (PMC9171144; doi:10.3389/fpls.2022.870438)
Supplement: Supplementary file 1 [file Data_Sheet_1.docx]

**Supplementary Table 1.** Percent of variance explained by each factor (Cultivar, Season, Rootstock and Irrigation) and the interactions (RxC, RxI and RxS) for the phenological and primary productivity variables, considering all available data. Only factors which explained a significant portion of the variance (p<0.05) are plotted. The percent variance explained by each factor is indicated using the value (%) and color. Darker colors explain a higher variance in each variable type.

| **Type** | **Variable** | **Cultivar (C)** | **Season (S)** | **Rootstock (R)** | **Irrigation (I)** | **RxC** | **RxI** | **RxS** |
| --- | --- | --- | --- | --- | --- | --- | --- | --- |
| **Phenology** | **Budburst** |  | 59.7 | 4.7 |  |  |  |  |
|  | **Flowering** | 6.6 | 25.1 | 2.1 |  |  |  |  |
|  | **Veraison** | 11.6 | 38.5 |  |  |  |  |  |
|  | **Days Bu-Ve** |  | 91.4 | 1.6 |  |  |  |  |
|  | **Harvest (22.5ºBrix)** |  | 33.6 | 6.3 | 12.4 |  |  |  |
| **Primary productivity** | **Yield** | 22 |  | 8.1 | 7.6 | 10.8 |  |  |
|  | **Nº Bunch per plant** |  | 28.7 | 3.8 |  | 10.4 |  |  |
|  | **Bunch Weight** | 38.7 | 14.3 |  | 4.1 |  |  |  |
|  | **Nº Berries per bunch** | 9.6 |  |  |  |  |  |  |
|  | **Berry Weight** | 45.6 | 7.3 |  | 2.7 |  |  | 4.3 |
|  | **Rachis Length** |  |  |  |  |  |  |  |
|  | **Rachis Weight** | 30.7 | 22.3 |  |  |  |  |  |
|  | **Caliber** | 39.7 |  | 6.4 | 6.8 |  |  | 4.7 |
|  | **Water Productivity** | 18.1 |  | 7.2 | 16.9 | 6.6 |  |  |
|  | **Pruning Weight** | 4.7 | 4.8 | 9.2 | 15 | 13.5 | 4.3 |  |
|  | **Trunk Circumference** | 7.4 | 17.5 | 15.6 | 9.6 | 22.7 | 4.5 |  |
|  | **Ravaz Index** |  | 10.5 | 6.4 | 8.2 |  |  |  |

**Supplementary Figure 1.** Precipitation (mm), minimum, and maximum air temperature (ºC) during the 2018-2019 (panel a) 2019-2020 (panel b) and 2020-2021 (panel c) seasons.


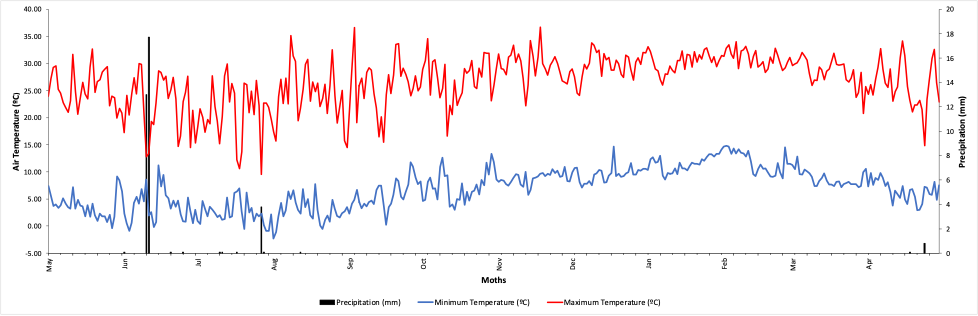


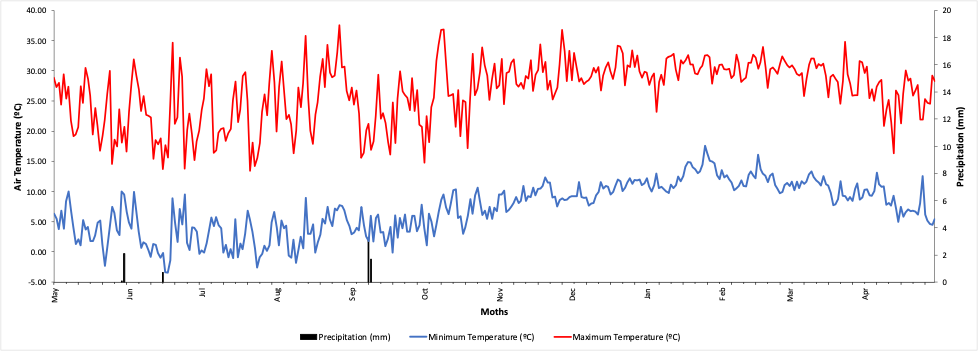


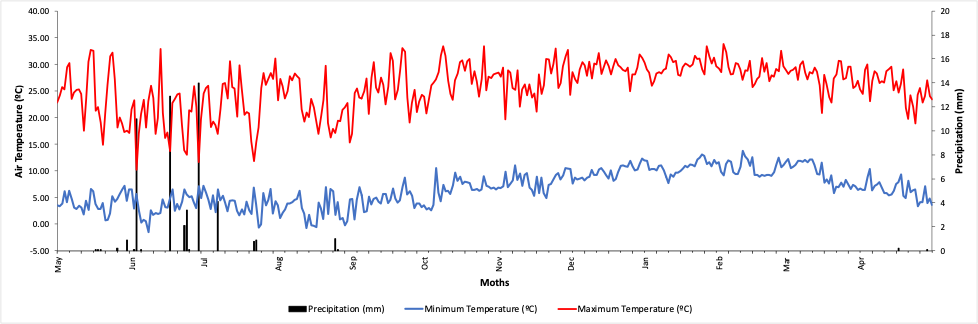


**Supplementary Figure 2.** Seasonal evolution of assimilation rate (A_n_) measured at midday in light-exposed leaves of Cabernet Sauvignon and grafted onto rootstocks R32 (square), R70 (diamond), 140Ru (triangle), and self-grafted (circle) under well-watered (T0) and sustained deficit irrigation (T1) conditions. Data are mean ± SE (n = 9).

**Supplementary Figure 3** Seasonal evolution of assimilation rate (A_n_) measured at midday in light-exposed leaves of Syrah and grafted onto rootstocks R32 (square), R70 (diamond), 140-Ru (triangle), and self-grafted (circle) under well-watered (T0) and sustained deficit irrigation (T1) conditions. Data are mean ± SE (n = 9).

**Supplementary Figure 4.** Seasonal evolution of stomatal conductance (g_s_) measured at midday in light-exposed leaves of Cabernet Sauvignon and grafted onto rootstocks R32 (square), R70 (diamond), 140-Ru (triangle), and self-grafted (circle) under well-watered (T0) and sustainable deficit irrigation (T1) conditions. Data are mean ± SE (n = 9).

**Supplementary Figure 5.** Seasonal evolution of stomatal conductance (g_s_) measured at midday in light-exposed leaves of Syrah and grafted onto rootstocks R32 (square), R70 (diamond), 140-Ru (triangle), and self-grafted (circle) under well-watered (T0) and sustained deficit irrigation (T1) conditions. Data are mean ± SE (n = 9).

**Supplementary Figure 6.** Seasonal evolution of stem water potential (Ψ_stem_) measured at midday in light-exposed leaves of Cabernet Sauvignon and grafted onto rootstocks R32 (square), R70 (diamond), 140-Ru (triangle), and self-grafted (circle) under well-watered (T0) and sustained deficit irrigation (T1) conditions. Data are mean ± SE (n = 3).

**Supplementary Figure 7** Seasonal evolution of stem water potential (Ψ_stem_) measured at midday in light-exposed leaves of Syrah and grafted onto rootstocks R32 (square), R70 (diamond), 140-Ru (triangle), and self-grafted (circle) under well-watered (T0) and sustainable deficit irrigation (T1) conditions. Data are mean ± SE (n = 3).
